# The Efficacy of the RME II System Compared with the Sander Bite-Jumping Appliance: A Retrospective Study

**DOI:** 10.3390/jcm14113700

**Published:** 2025-05-25

**Authors:** Mauro Lorusso, Michele Tepedino, Donatella Ferrara, Angela Pia Cazzolla, Fariba Esperouz, Rosa Esposito, Lucio Lo Russo, Domenico Ciavarella

**Affiliations:** 1 Department of Clinical and Experimental Medicine, Dental School of Foggia, University of Foggia, 71122 Foggia, Italy; donatella.ferrara@unifg.it (D.F.); angelapia.cazzolla@unifg.it (A.P.C.); fariba.esperouz@unifg.it (F.E.); rosa.esposito@unifg.it (R.E.); lucio.lorusso@unifg.it (L.L.R.); domenico.ciavarella@unifg.it (D.C.); 2 Department of Biotechnological and Applied Clinical Sciences, Dental School of L’Aquila, University of L’Aquila, 67100 L’Aquila, Italy; michele.tepedino@univaq.it

**Keywords:** Rapid Maxillary Expander (RME) II System, class II malocclusion, Sander appliance

## Abstract

**Objective:** This paper aimed to assess the effectiveness of the Rapid Maxillary Expander (RME) II System, compared to the Sander bite-jumping appliance (SBJ) and an untreated control group, in the treatment of Class II skeletal malocclusion in children. **Methods:** Thirty Class II patients treated with the RME II System (Group R) were compared to 30 patients treated with the SBJ (Group S) and 30 untreated Class II children (Group C). Cephalograms were analysed at the beginning of the study (T0) and at the end of the treatment (T1). Eight cephalometric parameters were evaluated: the divergence angle (SN-MP), ANB, lower face height (LFH), CO-GN, 1 + SN, IMPA, overjet, and overbite. The Shapiro–Wilk normality test was conducted to assess the distribution of the data. A *t*-test was then used for pairwise comparisons of the cephalometric measurements between T0 and T1. Differences among the groups were analysed using one-way ANOVA with Tukey’s post hoc correction. **Results:** ANOVA revealed a statistically significant difference for all analysed variables except 1 + SN. The post hoc Tukey’s test identified the following differences: SN-MP was 2.51° greater in Group S than in Group R, LFH was 5.46 mm greater in Group C than in Group R and 3.11 mm greater in Group S than in Group R, IMPA was 4.01° greater in Group S than in Group R, and overbite was 1.96 mm lower in Group S than in Group R. **Conclusions:** The RME II System provides better control of mandibular plane inclination and lower incisor proclination during the correction of Class II skeletal malocclusion. Both devices are effective in correcting Class II skeletal malocclusion.

## 1. Introduction

Class II malocclusion is a common disorder characterised by mandibular retrusion, labial incompetence, maxillary protrusion, narrow maxillary and mandibular dental arches, and compensatory labial tipping of the mandibular anterior teeth [1]. This malocclusion affects facial aesthetics and self-esteem, which can influence social and interpersonal relationships [2]. Although widespread, its prevalence varies geographically. The prevalence of Angle Class II malocclusion has been reported to be approximately 25% in the United States, Asia, and Europe, while the mean prevalence in Africa is 8.80% ± 10.36% [3]. In addition, approximately 80% of Caucasian patients with Class II malocclusion exhibit mandibular retrusion [4]. Consequently, the primary aim of treatment in these patients is mandibular advancement.

A patient’s craniofacial morphology is unique; therefore, the treatment plan must be individualised to achieve ideal occlusal, functional, and aesthetic outcomes. Furthermore, patients today are increasingly attentive to facial aesthetics, particularly their smiles [5,6]. Studies have shown that orthodontic treatment significantly impacts soft tissue changes, influencing both the overall appearance and function of the face. When crowding is present in both the upper and lower arches, therapy may involve the extraction of four premolars. However, if the lower arch is not crowded and there is adequate overjet, extractions may be limited to only the first or second upper premolars [7]. Extraction therapy may decrease the prominence of the upper and lower lips, reduce the interlabial gap, and increase the nasolabial angle [8,9]. Conversely, non-extraction therapy may result in protrusion of the upper and lower lips and a reduction in lip thickness, affecting smile aesthetics and overall soft tissue harmony [10]. Newer alternatives for Class II correction, such as distalisation of the upper molars, include appliances fixed with temporary anchorage devices or hybrid approaches combining clear aligners with skeletal anchorage [11,12].

During the growth period, therapy for Class II malocclusion caused by mandibular retrusion involves the use of functional appliances designed to stimulate mandibular growth and repositioning [13]. For this reason, assessing skeletal growth is essential to take advantage of peak pubertal growth. Several methods are available for assessing skeletal age, with the cervical vertebrae method being one of the most widely used [14]. The success of functional appliance therapy depends on various factors, including patient compliance with removable appliances, skeletal maturity, the severity of the underlying condition, and the timing of therapy [15,16]. However, Ahlgren observed that poor cooperation is one of the main reasons for the failure of functional therapy [17].

The management of Class II malocclusion is complex and requires a multifactorial diagnostic approach. Although the classification of malocclusions is a common clinical practice, it is important to recognise that relying solely on clinical and skeletal parameters can be limiting. Comprehensive assessment of the patient’s biotype and growth pattern is essential for accurate and individualised treatment planning. A thorough understanding of neuromuscular occlusal characteristics, together with the arch form associated with a specific craniofacial growth pattern, is essential for identifying the functional and morphological factors that contribute to the phenotypic manifestation of malocclusion [18,19].

Moreover, it is essential that clinicians not only understand the limitations of the appliances they employ, but also have the opportunity to select those that are clinically effective in addressing growth discrepancies across different spatial planes. Clinical studies provide critical insights into the multitude of factors requiring careful consideration and elucidate the inherent limitations and drawbacks associated with specific appliances [20].

Some of the most widely used functional removable devices include the Twin Block, the Bionator, and the Sander bite-jumping appliance (SBJ) [21]. The SBJ consists of two components: an upper unit and a lower unit, which, when the mouth is closed, position the jaw forward using two prongs. A systematic review by Santamaria-Villegas et al. [22] found that the SBJ is a more effective device for stimulating mandibular growth than the Twin Block, Bionator, Harvold Activator, and Frankel device. The Rapid Maxillary Expander (RME) II System is a functional appliance developed to treat Class II malocclusion with an associated maxillary width deficiency. This orthodontic device allows for the correction of both sagittal and transverse discrepancies in patients with Class II skeletal malocclusion. It is designed to minimise discomfort and reduce the undesirable effects commonly associated with other fixed orthodontic devices, due to their orthopedic impact on patients in active developmental stages [23]. Although less bulky and better tolerated by patients, this device still requires adequate patient cooperation.

Class II malocclusion, caused by mandibular retrusion, is frequently associated with transverse maxillary contraction [24,25]. Several authors have observed that correcting the transverse discrepancy can lead to repositioning of the mandible and a spontaneous improvement in the sagittal discrepancy [26,27]. Therefore, early intervention is needed to address the transverse defect in such cases. In the therapeutic management of Class II malocclusion, early intervention is often necessary, particularly in patients with a very increased overjet and labially inclined upper incisors, which are risk factors for trauma. In these cases, having a device to manage both the transverse and sagittal defects may be advantageous because it allows for the early resolution of the sagittal and transverse growth discrepancies, thereby addressing a complex malocclusion.

Palatal expansion can positively influence the sagittal positioning of the mandible in patients with mixed dentition [28]. In Class II malocclusion, in addition to skeletal and dental discrepancies, an important aspect to consider is the psychological discomfort associated with incisor protrusion and its impact on the facial appearance and profile. Typically, after early correction of the transverse discrepancy, clinicians wait before addressing the sagittal component. However, the ability to implement an early therapeutic approach could significantly enhance young patients’ self-esteem and confidence by improving their social interactions. This consideration is particularly important in the current social climate, where bullying remains a persistent challenge. To date, only one study has investigated the skeletal and dental effects of the RME II System [23]. The clinical significance of the present study lies in its aim to comprehensively evaluate these skeletal and dental effects, thereby providing clearer insight into the potential benefits and limitations of the device. The clinical relevance of the present study lies in the analysis of the skeletal and dental effects of the RME II System, aiming to provide a clearer understanding of the potential benefits and limitations of the device.

The aim of this study is to evaluate the dento-skeletal changes induced by the RME II System and the SBJ appliance in growing patients with Class II malocclusion. The null hypothesis states that there are no significant skeletal or dental differences between the two appliances at the end of treatment.

## 2. Material and Methods

This study was performed in accordance with the Strengthening the Reporting of Observational Studies in Epidemiology (STROBE) guidelines [29]. All methods specified in the study design complied with the Declaration of Helsinki and received approval from the Ethics Committee of the University. Retrospective collection and analysis of patient records were conducted with full anonymity, and signed authorisation was received from the parents of the participants.

The patients were selected based on the following inclusion criteria:

(1) Full Class II molar relationships;
(2) Overjet of >5 mm;
(3) Skeletal Class II division 1 malocclusion with mandibular retrusion assessed by cephalometric evaluation;
(4) Age between 9 and 13 years;
(5) Skeletal age between CS2 and CS3 according to the cervical vertebral maturation method.

The exclusion criteria were periodontal diseases, orofacial inflammatory conditions, tooth agenesis, congenital syndromes, previous orthodontic treatment, and temporomandibular joint disorders.

The power analysis (G*Power 3.1.9.2; Franz Faul, Universität Kiel, Germany) determined that a sample size of 28 patients per group was required to detect a large effect size of 0.4 [30] using one-way analysis of variance (ANOVA), with a significance level (α) of 0.05 and a statistical power (1 − β) of 0.90.

The study population was divided into three groups: Group R, which included subjects undergoing treatment with the RME II System; Group S, which included individuals who underwent treatment with the SBJ; and Group C, serving as the untreated control group. Groups R and S were retrospectively chosen from individuals treated at the Department of Orthodontics, University of Foggia, following a chronological selection process from March 2017 to October 2021. Treatment concluded once a Class I relationship of the molars and canines was attained. Patients participating in the study were instructed to wear the device for 16 h per day until the end of treatment. Group C was selected from the American Association of Orthodontists Foundation Craniofacial Growth Legacy Collection (https://www.aaoflegacycollection.org, accessed on 15 January 2024, Michigan Growth Study).

### 2.1. Group R

Group R comprised 30 patients (16 male and 14 female patients with a mean age of 9.1 years, standard deviation [SD] = 0.5), all treated with the RME II System. The average therapy duration was 12 months (range, 9–15 months).

**Figure 1:**
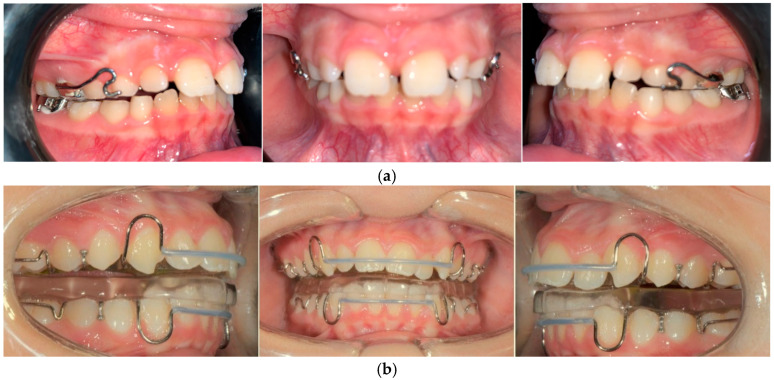
(**a**) Intraoral photos in frontal and lateral views of a patient treated with the RME II System; (**b**) intraoral photos in frontal and lateral views of a patient treated with the SBJ.

The RME II System is a functional orthodontic appliance incorporating a Hyrax expander anchored to the second primary molars. It features two rigid buccal arms extending toward the canines and a lower lingual arch with hooks for intermaxillary elastic attachment. Clinical images of the device are shown in Figure 1a. To promote progressive expansion, the device was adjusted at 21-day intervals, while participants were instructed to wear Class II elastics for 16 h per day. The elastic protocol included the use of 4.5 oz, 3/8″ elastics for four months, followed by 6 oz, 3/8″ elastics for one month, and concluding with 4 oz, 3/8″ elastics for two months. To ensure maximum traction efficiency, elastics were replaced daily.

### 2.2. Group S

Group S comprised 30 patients (15 male and 15 female patients with a mean age of 11.2 years, SD = 0.7 years) who were treated using the SBJ. The device consists of two plates: a lower plate with an inclined plane in the retroincisal area and an upper plate with a median expansion screw and two metal prongs. When the patient closes the mouth, the prongs slide along the inclined plane, moving the jaw forward. The construction bite was taken with the mandible advanced, positioning the incisors in a head-to-head relationship. Patients were instructed to wear the appliance for at least 16 h per day. The mean treatment time was 12 months (range, 8–14 months). Figure 1b shows clinical photographs of the device.

### 2.3. Group C

Group C comprised 30 patients (16 male and 14 female patients with a mean age of 9.9 years, SD = 1.4) who received no treatment. Patients were selected from the Michigan Medical Library and matched by age and sex to ensure comparability with the other two groups.

### 2.4. Cephalometric Analysis

**Figure 2:**
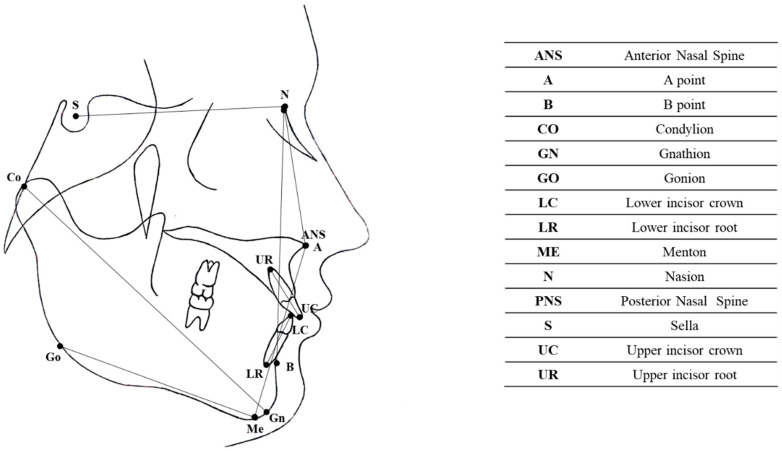
Cephalometric landmarks and reference lines.

The measurements were taken by a single operator on digitised cephalograms using a digital calliper (Screen Calliper version 4.0). The cephalometric analysis was performed by the same operator, who had received extensive training in electronic cephalometric analysis, using Dolphin Imaging 11.0 software (Chatsworth, CA, USA). The cephalometric measurements were taken blindly; the orthodontist who conducted the analyses was not involved in the treatment of the patients, and the data were anonymised before the analyses were performed. The following cephalometric skeletal and dental variables were analysed: SN-MP, ANB, lower face height (LFH), CO-GN, 1 + SN, IMPA, overjet, and overbite. The landmarks and reference lines used in the cephalometric analysis are shown in Figure 2 and listed in Table 1.

### 2.5. Statistical Analysis

To minimise random errors, cephalometric and dental measurements were taken twice. The random error for each measurement was calculated using Dahlberg’s formula (S = ∑ d^2^/2N), where d represents the difference between the first and second measurements and N is the number of radiographs evaluated [31,32]. The random error ranged from 0.22 to 0.35 mm for linear measurements and from 0.32° to 0.46° for angular measurements.

Normality of the data was tested using the Shapiro–Wilk method. Because the variables were normally distributed, a paired *t*-test (Table 2) was used for pairwise comparisons of the cephalometric measurements taken at T0 (pre-treatment) and T1 (post-treatment) within each group. The differences among the three groups were evaluated using one-way ANOVA for the T1–T0 difference of each variable, followed by Tukey’s post hoc test. Statistical significance was set as *p* < 0.05.

## 3. Results

Table 2 presents the *t*-test results at T0 and T1 for each group. The one-way ANOVA results (Table 3) revealed statistically significant differences across all analysed variables, except for 1 + SN. Post hoc analysis using Tukey’s test (Table 4) revealed the following statistically significant differences:

**Table 2:** *t*-test comparing cephalometric variables at T0 and T1 among the three groups (statistically significant correlations are in bold).

	Group R					Group S					Group C				
	T0		T1		*p*	T0		T1		*p*	T0		T1		*p*
	Mean	Std Dev.	Mean	Std Dev.		Mean	Std Dev.	Mean	Std Dev.		Mean	Std Dev.	Mean	Std Dev.	
SN-MP	34.41	4.23	32.87	4.51	0.001	33.92	4.24	32.08	4.61	0.55	34.21	3.76	33.76	3.95	0.05
LFH	65.29	6.21	64.33	4.71	0.396	61.94	4.98	64.80	5.31	0.001	64.11	9.24	63	8.80	0.372
CO-GN	101.7	4.79	106.6	4.29	0.001	100.7	6.41	105.1	6.31	0.001	103.9	12.6	103.4	13.93	0.4
1 + SN	106.4	7.18	103.8	5.29	0.031	106.20	5.65	103.74	6.04	0.03	107	4.78	105.9	5.78	0.001
IMPA	95.33	6.37	96.2	6.19	0.863	93.77	8.1	97.48	8.46	0.001	90.85	6.43	91.18	7.18	0.354
Overbite	1.61	1.54	2.84	1.26	0.001	2.73	1.94	1.85	1.03	0	3.37	3.16	3.72	1.40	0.002
Overjet	6.81	1.78	3.34	1.24	0.001	7.73	2.17	3.81	1.12	0.001	6.31	1.55	5.5	2.51	0.005
ANB	5.57	1.11	2.78	0.89	0.002	5.84	1.16	3.96	1.02	0.001	5.61	1.5	5.2	1.01	0.22

(1) The divergence angle was 1.81° greater in the control group than in the RME II System group, and 2.51° greater in the Sander group compared to the RME II System group;
(2) Lower facial height (LFH) was 5.46 mm greater in the control group than in the RME II System group, and 3.11 mm greater in the Sander group compared to the RME II System group;
(3) The mandibular length (CO-GN) was 4.33 mm shorter in the control group than in the RME II System group and 6.08 mm shorter in the control group compared to the Sander group;
(4) Lower incisor inclination (IMPA) was 3.34° lower in the control group than in the Sander group and 4.01° greater in the Sander group compared to the RME II System group;
(5) The overbite was 3.07 mm greater in the control group than in the Sander group and 1.96 mm lower in the Sander group compared to the RME II System group;
(6) The overjet was 3.59 mm greater in the control group than in the RME II System group and 4.88 mm greater in the control group compared to the Sander group;
(7) The ANB angle was 2.15° greater in the control group than in the RME II System group and 1.38° greater in the Sander group compared to the RME II System group.

Therefore, the null hypothesis was rejected.

## 4. Discussion

The study aimed to assess the dentoskeletal changes induced by the RME II System and the SBJ in Class II malocclusion, compared with an untreated control group. The bite-jumping device was conceptualised by Kingsley in 1879, who made significant contributions to the development of functional orthodontics [33]. Building on the original design conceptualised by Kingsley, Sander developed a double-block mandibular advancement device that incorporated the principles of bite jumping [21]. Since 1979, this device has been widely used and is considered one of the most effective mandibular functional appliances.

In this paper, an increase in the divergence angle (Sn-GOMe) and LFH was detected in patients treated with the SBJ compared with those treated with the RME II System. By contrast, Martina et al. [34] did not observe any variations in the divergence angle after treatment with the SBJ. It is important to highlight that at time T0, the divergence angle was greater in patients in the RME II System group. However, patients treated with the RME II System showed greater control of divergence. This result is consistent with the findings of a previous study in which patients treated with the RME II System were compared to those treated with the Herbst appliance [23]. This aspect is particularly important because when clockwise rotation of the mandible occurs, the B-point moves backward, thereby worsening the malocclusion.

With regard to mandibular length, an increase in the Co-Gn mandibular length after therapy was observed in both Group S and Group R in the present study. Specifically, the mandibular length was 4.83 mm greater in Group R than in Group C and 6.08 mm greater in Group S than in Group C. Thus, in both groups, the devices demonstrated an orthopaedic effect that promoted mandibular growth. These findings are in agreement with Sambale et al. [35], who observed an increase in jaw length following SBJ therapy. In a meta-analysis by Santamaria-Villegas et al. [22], the SBJ resulted in greater mandibular growth than other functional devices, such as the Twin Block and Bionator. By contrast, Tepedino et al. [36] observed greater mandibular growth in a group of subjects managed with the Twin Block compared to those treated with the SBJ. These data are in line with those of Martina et al. [34], who observed an increase in mandibular length after SBJ therapy. However, it is interesting to note that the comparison of T1 − T0 differences between the two devices showed no significant variations. Both devices resulted in increased mandibular growth at the end of therapy.

In the current study, a notable difference was observed in the inclination of the lower incisors between Groups S and R. In particular, an increase in IMPA was found in the group of patients treated with the SBJ. Therefore, in these patients, therapy resulted in proclination of the lower incisors. These results are consistent with Rizio et al. [34], who observed an approximately 3° greater proclination of the lower incisors in patients treated with the SBJ than in controls. By contrast, Sander et al. [37] did not observe any changes in the inclination of the lower incisors following SBJ therapy, while Gazzani et al. [38] observed a slight inclination of the lower incisors (1.3°) after SBJ therapy. It appears that the results in the literature are conflicting. However, as evidenced by the results of this study, the RME II System allows for better control over the positioning of the lower incisors than the SBJ. This aspect is of paramount importance because the tilting of the incisors reduces the overjet and, consequently, limits the space for mandibular repositioning.

A significant reduction in overjet was observed in both Groups R and S compared with the control group. Consistent with the findings of this research, Faccioni et al. [39] reported a notable decrease in overjet among patients treated with the SBJ.

Regarding the ANB angle, a comparison between the groups showed that this angle was greater in Group S than in Group R. Consequently, there was a greater reduction in this angle among patients treated with the RME II System. Sander et al. [37] observed a significant decrease in the ANB angle in subjects treated with SBJ compared to the control group. Consistent with the findings of the current research, Sambale et al. [35] also observed a notable decrease in the ANB angle in patients treated with the SBJ.

Finally, regarding overbite, a reduction was observed in the SBJ group compared with Group R. This reduction can be explained by the increase in the divergence angle and LFH observed at the end of therapy in patients treated with the SBJ.

The findings of this paper indicate that both appliances are effective in correcting Class II malocclusion caused by mandibular retrusion. The differences observed between the two devices are attributable to their distinct designs and mechanisms of action. Better divergence control and reduced proclination of the lower incisors were detected in subjects undergoing treatment with the RME II System. This aspect is particularly important in the treatment of hyperdivergent patients because increased divergence may exacerbate malocclusion. Furthermore, maintaining greater control over the inclination of the lower incisors is crucial because incisor proclination reduces the overjet, thereby limiting the available space for mandibular advancement.

### Limitations of the Study

This study has several limitations. Because of the retrospective nature of the study, the effectiveness of the devices may have been overestimated. Additionally, the retrospective design could have introduced selection bias; however, strict inclusion criteria were applied to mitigate this risk. Finally, the lack of follow-up data limited the assessment of long-term outcomes. Further studies should involve a larger participant pool and include a longer follow-up.

## 5. Conclusions

The RME II System appears to provide better control over divergence and lower incisor inclination. The SBJ appears to have a greater effect on mandibular growth; however, it may lead to increased inclination of the lower incisors, reduced divergence control, and consequently, a decreased overbite. Clinicians are strongly advised to carefully evaluate individual patient characteristics and compliance potential before selecting the appropriate appliance. Device selection must take into account the patient’s craniofacial characteristics and the specific outcomes the clinician aims to improve in order to maximise the therapeutic effects of these devices. Proactive monitoring throughout treatment is essential to mitigate adverse effects, optimise skeletal and dental outcomes, and ensure the most effective management of therapy. Based on the results of the present study, both devices were effective in the treatment of Class II malocclusion.

## Figures and Tables

**Table 1:** Cephalometric measurements.

Measurement	Description
***Skeletal measurements***	
SN-MP	Angle between the sella–nasion (SN) line and the mandibular plane (MP)
ANB	Angle between the N–A line and N–B line
LFH (lower face height)	Distance between the anterior nasal spine (ANS) and the menton (ME)
MANDIBULAR LENGTH (CO-GN)	Distance between the condylion and the gnathion
***Dental measurements***	
1 + SN	Angle between the upper incisor and the sella–nasion line
IMPA	Angle between the lower incisor and the mandibular plane (GO–ME)
OVERJET	Horizontal distance between the incisal border of the upper and lower incisors
OVERBITE	Vertical distance between the incisal border of the upper and lower incisors

**Table 3:** One-way ANOVA comparing variables between groups.

		Sum of Squares	df	Mean Square	F	*p*
SN-MP	Between groups	100,987	2	50,494	16,378	0.001 **
	Within groups	268,216	88	3083		
	Total	369,204	90			
Lower Face Height	Between groups	451,207	2	225,603	19,052	0.001 **
	Within groups	1,030,216	88	11,842		
	Total	1,481,423	90			
CO-GN	Between groups	589,342	2	294,671	15,295	0.001 **
	Within groups	1,676,171	88	19,266		
	Total	2,265,513	90			
1 + SN	Between groups	212,339	2	106,169	2299	0.106
	Within groups	4,018,160	88	46,186		
	Total	4,230,499	90			
IMPA	Between groups	277,328	2	138,664	8244	0.001 **
	Within groups	1,463,357	88	16,820		
	Total	1,740,685	90			
Overbite	Between groups	144,986	2	72,493	17,797	0.001 **
	Within groups	354,379	88	4073		
	Total	499,365	90			
Overjet	Between groups	384,078	2	192,039	42,226	0.001 **
	Within groups	395,669	88	4548		
	Total	779,746	90			
ANB	Between groups	71,239	2	35,619	13,691	0.001 **
	Within groups	226,353	88	2602		
	Total	297,592	90			

** *p* < 0.01.

**Table 4:** Tukey’s post hoc test.

Dependent Variable	(I) Group	(J) Group	Mean Difference (I–J)	Std Error	*p*	95% Confidence Interval	
						Lower Bound	Upper Bound
SN-MP	C	R	1.81 *	0.453	0.001	0.734	2.896
	C	S	−0.698	0.453	0.277	−1.779	0.382
	S	R	2.51 *	0.453	0.001	1.432	3.594
Lower Face Height	C	R	5.46 *	0.888	0.001	3.348	7.585
	C	S	2.35	0.888	0.026	0.235	4.472
	S	R	3.11 *	0.888	0.002	0.994	5.232
CO-GN	C	R	−4.83 *	1.133	0.001	1.632	7.037
	C	S	−6.08 *	1.133	0.001	3385	8.790
	S	R	1.75	1.133	0.274	4.455	0.94
1 + SN	C	R	3.25	1.754	0.159	−0.934	7.434
	C	S	3.26	1.754	0.156	−0.917	7.450
	S	R	−0.01	1.754	1.001	−4.208	4.167
IMPA	C	R	0.66	1.058	0.807	−1.864	3.186
	C	S	−3.34 *	1.058	0.006	−5.874	−0.824
	S	R	4.01 *	1.058	0.001	1.485	6.535
Overbite	C	R	1.11	0.521	0.09	−0.132	2.352
	C	S	3.07 *	0.521	0.001	1.8274	4.312
	S	R	−1.96 *	0.521	0.001	−3.202	−0.717
Overjet	C	R	3.59 *	0.550	0.001	2.277	4.903
	C	S	4.88 *	0.550	0.001	3.570	6.196
	S	R	−1.29	0.550	0.054	−2.606	0.019
ANB	C	R	2.15 *	0.416	0.001	1.156	3.143
	C	S	0.76	0.416	0.162	−0.226	1.759
	S	R	1.38 *	0.416	0.004	0.390	2.376

* *p* < 0.05.

## Data Availability

The data presented in this study are available on request from the corresponding author due to privacy reasons.
